# Immunomodulatory therapy for sepsis: from pathophysiological mechanisms to precision treatment

**DOI:** 10.3389/fcimb.2025.1746254

**Published:** 2026-02-11

**Authors:** Yiting Xiao, Liyun Xu, Yuan Jiang, Qian Wang, Jie Deng, Zixiang Luo, Wenchao Xie, Caihong Ye, Zhangrui Zeng

**Affiliations:** 1 Department of Laboratory Medicine, The Affiliated Hospital, Southwest Medical University, Luzhou, China; 2 Sichuan Province Engineering Technology Research Center of Molecular Diagnosis of Clinical Diseases, Luzhou, China; 3 Molecular Diagnosis of Clinical Diseases Key Laboratory of Luzhou, Luzhou, China

**Keywords:** biomarker, cytokine storm, endotype, immunomodulation, immunosuppression, precision medicine, sepsis

## Abstract

Sepsis is a life-threatening syndrome marked by immune dysregulation, progressing from hyperinflammation to immunosuppression. The translation of immunomodulatory therapies has been hampered by the disease’s extreme heterogeneity. This review synthesizes current progress and future perspectives in sepsis immunotherapy. We outline key immunopathological mechanisms and critically discuss evolving diagnostic tools, including dynamic biomarker monitoring and immune endotyping for personalized management. We then highlight novel therapeutic targets and explore how integrating single-cell technologies, dynamic profiling, and machine learning can guide stage-specific, precision treatment. Ultimately, a precision medicine framework combining multi-omics data with advanced bioengineering may offer new avenues to overcome the therapeutic impasse in sepsis.

## 1 Introduction

Sepsis remains a leading cause of mortality in intensive care units worldwide, representing a profound public health challenge characterized by dysregulated host responses to infection and life-threatening organ dysfunction. Despite decades of research and advances in supportive care, mortality rates remain unacceptably high, underscoring the limitations of a generalized treatment approach. The core obstacle lies in the syndrome’s extreme heterogeneity; the clinical course can vary dramatically between patients, spanning an early hyperinflammatory “cytokine storm” to a late-stage immunosuppressive state, often with considerable overlap. This pathophysiological complexity has led to the repeated failure of monotherapeutic immunomodulatory strategies (e.g., anti-TNF-α, anti-IL-1) in pivotal clinical trials over the past decades, which have historically treated sepsis as a single entity rather than a spectrum of immunological disorders. Consequently, the field is undergoing a paradigm shift from a “one-size-fits-all” model toward precision medicine, aiming to tailor immunomodulatory interventions to a patient’s individual immune phenotype and the temporal stage of their illness. This review synthesizes the current understanding of sepsis immunopathology, from fundamental mechanisms to diagnostic innovations, and explores how emerging technologies—including dynamic immune monitoring, single-cell analytics, and machine learning—are paving the way for the development of targeted, stage-specific, and ultimately effective immunotherapeutic strategies for this complex syndrome.

What is new in this review? Unlike previous reviews that focus on a single dimension—such as either immunopathological mechanisms or therapeutic interventions—this work presents an integrated framework that connects evolving mechanistic insights (e.g., the TLR4/PTEN axis, metabolic reprogramming) with actionable precision medicine strategies (e.g., dynamic endotyping, machine learning–guided therapy). Particular emphasis is placed on the critical challenge of patient heterogeneity and the importance of pathogen-type-specific considerations in both diagnosis and treatment. Moreover, emerging approaches such as CAR-macrophages are discussed within the context of the unique temporal dynamics of sepsis. Collectively, this synthesis aims to bridge the persistent gap between mechanistic discovery and clinical translation.

### 1.1 Literature search strategy

To ensure an up-to-date synthesis, a literature search was conducted in PubMed and Web of Science covering the period from 2018 to 2024. The search employed key terms including “sepsis,” “immunotherapy,” “precision medicine,” “endotype,” “biomarker,” “TLR4,” “cytokine storm,” “immune checkpoint,” and “CAR macrophage.” Priority was given to high-impact original studies, landmark clinical trials, and authoritative review articles. This narrative review is designed to integrate these findings within a novel framework that connects mechanistic understanding to precision therapeutic strategies, rather than to perform a quantitative systematic analysis.

## 2 Advances in the immunopathological mechanisms of sepsis

### 2.1 Innate immune dysregulation and the pivotal role of the TLR4/PTEN signaling axis

**Figure 1:**
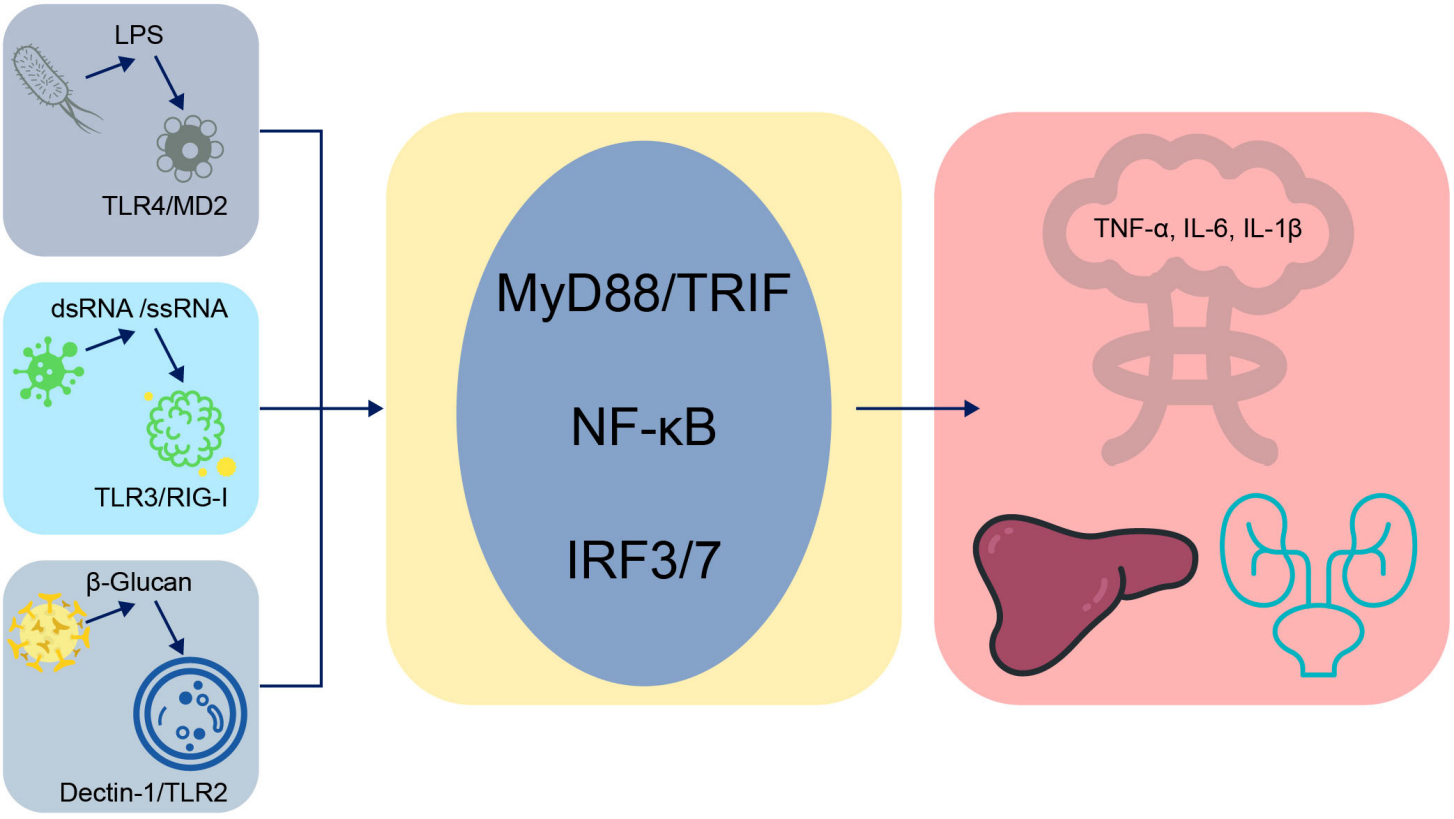
Development and therapeutic workflow of Chimeric Antigen Receptor Macrophage (CAR-M) therapy for sepsis. This flowchart illustrates the conceptual and technical workflow underlying the application of chimeric antigen receptor–macrophage (CAR-M) cell therapy. The process involves several sequential stages: (1) sourcing precursor cells, either autologous monocytes obtained from the patient’s peripheral blood or induced pluripotent stem cells (iPSCs); (2) genetically engineering these cells to express a chimeric antigen receptor (CAR) composed of an extracellular antigen-recognition domain for pathogen-specific targeting and intracellular signaling domains for effector activation; (3) expanding the successfully transduced CAR-M cells ex vivo to achieve a clinically relevant dose; (4) reinfusing the expanded cellular product into the septic patient; and (5) enabling the infused CAR-M cells to exploit their intrinsic phagocytic activity and tissue-homing properties to identify, internalize, and eliminate targeted pathogens (e.g., bacteria, fungi). Beyond direct pathogen clearance, engineered CAR-Ms can modulate the dysregulated immune microenvironment through cytokine secretion, thereby exerting both antimicrobial and immunomodulatory effects. This approach exemplifies a precision bioengineering paradigm aimed at delivering targeted therapy while potentially mitigating nonspecific immunopathology.

Sepsis initiates with excessive innate immune activation, primarily via the Toll-like receptor 4 (TLR4) pathway. TLR4 recognizes pathogen (e.g., LPS) and damage-associated molecular patterns, triggering downstream signaling that activates transcription factors like NF-κB and IRFs. This leads to a massive release of pro-inflammatory cytokines (e.g., TNF-α, IL-6) and interferons, driving the initial “cytokine storm” and subsequent organ dysfunction (Wang et al., 2025). The phosphatase and tensin homolog (PTEN) acts as a crucial intrinsic brake on this hyperactive signaling. PTEN, a lipid phosphatase, primarily dephosphorylates phosphatidylinositol (3,4,5)-trisphosphate (PIP3), the key product of PI3K activation. By antagonizing PI3K/Akt signaling, PTEN negatively regulates TLR4-driven inflammatory responses and helps maintain immune homeostasis. Recent studies further reveal that the endogenous alarmins S100A8/A9 serve as DAMPs that exacerbate inflammation via TLR4 binding. In animal models, genetic inhibition of the S100A8/A9-TLR4 axis improved sepsis outcomes, potentially by reducing platelet pyroptosis (Zhou et al., 2023). Critically, PTEN loss or impairment, mediated by oxidative stress, transcriptional downregulation, or microRNAs in sepsis, removes this regulatory brake. This amplifies PI3K/Akt/mTOR signaling, leading to enhanced NF-κB activity and correlating with the severity of immune dysregulation and poor outcomes in sepsis. This delicate balance establishes the TLR4/PTEN axis as a finely tuned immunoregulatory network: a shift toward TLR4 dominance drives hyperinflammation, while its dysregulation also contributes to the later immunosuppressive phase through mechanisms such as immune cell exhaustion. The disruption of this axis, therefore, plays a pivotal role throughout the immunopathogenesis of sepsis (Wang et al., 2025; Zhou et al., 2023) (see **Figure 1**), and its downstream effects, particularly through the PI3K/Akt/mTOR pathway, critically contribute to the metabolic reprogramming and immune cell dysfunction observed in later stages (see Section 2.3). Beyond protein-level regulation, microRNAs (miRNAs) have emerged as critical post-transcriptional modulators of the host response in sepsis. In particular, miRNAs such as miR-21 and miR-146a target key components of the TLR4 signaling pathway, thereby fine-tuning the inflammatory response. Conversely, activation of TLR4 can modify cellular miRNA expression profiles, establishing regulatory feedback loops. This dynamic miRNA–TLR4 interaction not only influences the magnitude of inflammation but also possesses prognostic significance, as specific miRNA signatures have been correlated with sepsis-related mortality. These findings underscore their potential utility as both biomarkers and therapeutic targets.

### 2.2 The molecular basis for cytokine storm induction

The cytokine storm is a defining immunopathological hallmark of early sepsis, characterized by a massive and rapid release of pro-inflammatory mediators that causes collateral tissue injury. This process involves complex positive feedback loops and extensive cross-talk among signaling pathways. The cytokine storm is amplified by interconnected pathways. Activation of the indoleamine 2,3-dioxygenase 1 (IDO1) pathway depletes tryptophan, generating kynurenine metabolites that, via the AHR-CYP1A1 axis, fuel further cytokine release and immune cell death. This axis synergizes with classical inflammatory signaling (e.g., NF-κB, STAT3) to create a self-perpetuating inflammatory loop (Chen et al., 2025). A stable tripartite feedback loop among the cytokine storm, inflammatory mediators (e.g., IL-6, TNF-α) (see **Figure 1**), and the IDO1-AHR-CYP1A1 axis renders the inflammatory response self-perpetuating and resistant to resolution (Pugin et al., 2021; Wang et al., 2023). Within this network, tumor necrosis factor-alpha (TNF-α) serves as the earliest and most potent initiator of inflammation, triggering downstream cascades. Monocyte chemoattractant protein-1 (MCP-1) amplifies the response by recruiting monocytes and macrophages to sites of infection. Interleukin-6 (IL-6), a pleiotropic cytokine that induces acute-phase protein synthesis and activates lymphocytes, is a major mediator of the cytokine storm and a biomarker of sepsis severity. The interplay among these cytokines represents a critical nexus for understanding early sepsis pathophysiology and for developing targeted interventions (Pugin et al., 2021; Yang et al., 2025).

### 2.3 Metabolic reprogramming in the immunosuppressive phase

As sepsis progresses, the immune system often transitions from early hyperinflammation to a later immunosuppressive phase commonly referred to as compensatory anti-inflammatory response syndrome (CARS) or immunoparalysis. This state is characterized by persistently elevated anti-inflammatory cytokines (e.g., IL-10, TGF-β); impaired innate immune cell functions (e.g., reduced phagocytosis and antigen presentation by neutrophils and monocytes/macrophages); apoptosis or exhaustion of adaptive immune cells (T and B lymphocytes); and the expansion of immunosuppressive populations such as myeloid-derived suppressor cells (MDSCs) and regulatory T cells (Tregs), which further dampen immune activity (Zhang et al., 2024) (McBride et al., 2020). This immunosuppression is driven by metabolic reprogramming, stemming from upstream dysregulation (e.g., PTEN/PI3K/Akt/mTOR). In sepsis, hypoxic and nutrient-deprived conditions disrupt energy metabolism, impairing the balance between anabolic (mTOR) and catabolic (AMPK) signaling. This metabolic dysfunction directly cripples immune cell function (Wiersinga and van der Poll, 2025). Additionally, immune checkpoint molecules such as programmed cell death protein 1 (PD-1) and its ligand PD-L1 are upregulated on T cells and other immune cells, delivering inhibitory signals that induce T cell exhaustion, which is a key mechanism of sepsis-induced immunosuppression (Zhong and Yin, 2023). Epigenetic regulation also plays a role, as emerging evidence has revealed that RNA modifications (e.g., m^6^A methylation) can fine-tune the expression of immune-related genes by affecting mRNA stability and translation efficiency, thereby significantly shaping septic immunosuppression and presenting a promising novel therapeutic target (Vignon et al., 2020). The key cellular and molecular features of this immunosuppressive phase are outlined in **Table 1**. In summary, late-stage sepsis immunosuppression serves an adaptive yet ultimately deleterious process driven by metabolic dysregulation, immune checkpoint activation, and epigenetic remodeling. This culminates in impaired host defense, heightened vulnerability to secondary infections, and persistent organ dysfunction, comprising a vicious cycle of immune and metabolic collapse (McBride et al., 2021; Wang et al., 2023; Zhang et al., 2024).

**Table 1:** Key characteristics of the immunosuppressive phase in sepsis.

Category	Key features/alterations
Cellular dynamics	• Lymphocyte apoptosis and exhaustion • Expansion of myeloid-derived suppressor cells (MDSCs) and regulatory T cells (Tregs) • Functional impairment of monocytes/macrophages (e.g., reduced HLA-DR expression)
Molecular & metabolic reprogramming	• Shift in immune cell metabolism • Dysregulation of energy sensing and anabolic pathways • Epigenetic modifications
Functional consequences	• Inability to clear primary infection • High susceptibility to secondary infections • Persistence of organ dysfunction

The immunopathological mechanisms detailed above—from the initial dysregulation of the TLR4/PTEN axis and the ensuing cytokine storm to the subsequent metabolic reprogramming and immunosuppression—collectively underscore the profound heterogeneity of sepsis. This biological complexity translates directly into the clinical challenge of reliably identifying and stratifying septic patients. Consequently, the evolving landscape of sepsis diagnostic criteria, represents an ongoing effort to reconcile these multifaceted pathophysiological realities with the pragmatic need for timely, accurate, and prognostically useful clinical tools.

## 3 Evolution and controversies in sepsis diagnostic criteria

### 3.1 Clinical validation: comparing the sepsis-2 and sepsis-3 criteria

The diagnostic criteria for sepsis have evolved, with Sepsis-3 (2016) representing a paradigm shift by defining sepsis as life-threatening organ dysfunction due to a dysregulated host response to infection, and recommending the SOFA score (increase ≥2 points) for diagnosis (Martin-Loeches et al., 2024). This transition marked a shift in emphasis from detecting inflammation alone (as with SIRS, which is sensitive but lacks specificity) toward identifying infection-associated organ dysfunction, thereby aligning diagnostic criteria more closely with the core pathophysiology of sepsis in the form of host immune dysregulation. Compared with the broader Sepsis-2 criteria, Sepsis-3 improves diagnostic specificity by ensuring that identified patients are at substantial risk of infection-induced organ failure (Martin-Loeches et al., 2024). This emphasizes organ dysfunction over inflammation alone, improving specificity but potentially reducing sensitivity for early detection in non-ICU settings (Yuan et al., 2024). Consequently, there is interest in combining clinical scores (e.g., NEWS2, qSOFA) with biomarkers like procalcitonin for earlier risk stratification, though these require further validation (Martin-Loeches et al., 2024). A direct comparison of the Sepsis-2 and Sepsis-3 criteria is summarized in **Table 2**.

**Table 2:** Comparison of the Sepsis-2 and Sepsis-3 diagnostic criteria (Rincon et al., 2023; Stern et al., 2023).

Criteria aspect	Sepsis-2	Sepsis-3
Core definition	Infection + Systemic Inflammatory Response Syndrome (SIRS)	Life-threatening organ dysfunction caused by a dysregulated host response to infection
Primary diagnostic tool	SIRS Criteria (≥2 criteria)	Increase in SOFA score ≥ 2 points from baseline
Advantage	High sensitivity for early screening	High specificity for identifying patients at high risk of mortality
Limitation	Low specificity, includes non-infectious inflammation	Potential delay in recognition in non-ICU settings due to a focus on organ dysfunction

### 3.2 Dynamic biomarker monitoring strategies

Dynamic biomarker monitoring complements clinical criteria. Procalcitonin (PCT) is elevated in bacterial sepsis and helps distinguish bacterial from viral infections (Zaki et al., 2024). C-reactive protein (CRP) is sensitive but non-specific (Yuan et al., 2024). No single biomarker is sufficient; combined panels and trend monitoring are increasingly used (Marin et al., 2024). Conventional white blood cell counts and blood differential tests remain widely used due to their accessibility, but are non-specific, as sepsis can manifest with either leukocytosis or leukopenia. In-depth analyses of neutrophil phenotypes that include differentiation among hyperinflammatory, hypoinflammatory, and intermediate subsets may ultimately provide more precise insights into immune status and disease severity (Yang et al., 2024). Importantly, the immunopathology of sepsis is extraordinarily complex, and no single biomarker can comprehensively reflect its dynamic progression or fully differentiate it from other systemic inflammatory disorders with similar presentations (e.g., severe pancreatitis, major trauma) (Póvoa et al., 2023). As such, clinical practice increasingly favors combined biomarker panels, with an emphasis on monitoring temporal trends rather than isolated values. Tracking dynamic biomarker trajectories (e.g., PCT, CRP) offers superior predictive value for early host-response dysregulation compared to single measurements (Barichello et al., 2022; Pugin et al., 2021).

### 3.3 Prognostic value of immune phenotype-based classification

High-dimensional technologies (e.g., mass cytometry, transcriptomics) enable immune endotyping, stratifying patients into hyperinflammatory (excessive cytokine release) and immunosuppressive (lymphopenia, exhaustion) endotypes. These endotypes predict prognosis and therapy response; for example, anti-inflammatory interventions may help hyperinflammatory but harm immunosuppressed patients (Bode et al., 2023; Pokhrel et al., 2021). Longitudinal monitoring of lymphocyte subsets and bioinformatic analyses of immune infiltration further refine prognostic assessment (Wang et al., 2023) (Al Gharaibeh et al., 2025; Zhou et al., 2023). Collectively, immune phenotyping provides a foundation for precision medicine in sepsis, facilitating individualized therapeutic decisions tailored to each patient’s immune landscape. However, translating these advanced phenotyping platforms into rapid, standardized, and clinically deployable bedside assays remains a significant challenge (He et al., 2020; Wang et al., 2023).

## 4 Targets for immunomodulatory therapy

### 4.1 Interventional strategies targeting inflammatory factors

Given the central role of cytokine storm activity in the early pathophysiology of sepsis, directly targeting key inflammatory cytokines remains a major therapeutic avenue of interest. TNF-α, as an upstream initiator of the inflammatory cascade, has remained a primary focus of these treatment efforts. Preclinical studies indicate that inhibiting TNF-α can alleviate sepsis-induced organ dysfunction, in part by mitigating TNF-α-associated mitochondrial injury through pathways like AMPK-Sirt3 (Wang et al., 2023). Mechanistically, TNF-α-induced mitochondrial dysfunction is a pivotal event in cellular injury, with the AMPK-Sirt3 signaling pathway serving as a key regulatory axis. AMPK activators (e.g., A769662) can partially reverse TNF-α-mediated mitochondrial impairment by upregulating this pathway, offering a potential strategy for organ protection (Wang et al., 2023). Despite promising preclinical results, translating anti-TNF-α therapies into clinical benefit has proven difficult. Numerous trials evaluating anti-TNF-α agents in septic patients have failed to demonstrate significant survival benefits. The lack of efficacy likely reflects the fundamental challenges of sepsis trial design: pronounced patient heterogeneity, the narrow and elusive optimal therapeutic window within a rapidly evolving immune response, and the redundancy within the cytokine network which limits the impact of single-cytokine blockade (Bode et al., 2023). These failures underscore the necessity for patient stratification and staged treatment approaches. Future approaches should emphasize identifying patient subgroups most likely to benefit from anticytokine therapy through precise immune endotyping and the administration of appropriate interventions within a carefully defined temporal window. It is important to note that TNF-α is a key downstream effector of the hyperactive TLR4 signaling pathway. Therefore, anti-TNF-α strategies can be viewed as an attempt to mitigate the consequences of this upstream dysregulation. However, the limited success of these strategies also highlights the challenge of targeting a single, redundant node within a complex network, shifting focus toward more upstream regulators such as the TLR4/PTEN axis or toward precise patient endotyping.

### 4.2 Immune checkpoint modulators

During the immunosuppressive phase of sepsis, co-inhibitory immune checkpoint molecules (e.g., PD-1, CTLA-4, TIM-3) are markedly upregulated on T cells and other immune cells. Engagement of these receptors transmits inhibitory signals, leading to T cell exhaustion and immunoparalysis, thereby impairing the host’s ability to clear primary and secondary infections. Consequently, the use of immune checkpoint inhibitors (ICIs) to “release the brakes” on the immune system and restore T cell function holds promise as a viable therapeutic strategy. Clinical observations have shown that cancer patients receiving ICIs (e.g., PD-1 inhibitors) who develop sepsis may experience better survival outcomes than septic patients not receiving such therapies, indirectly supporting this approach’s potential. Case reports and small clinical studies have explored the off-label use of ICIs (e.g., nivolumab) in refractory sepsis, noting improvements in immune parameters and clinical status in some patients (Yu et al., 2025). Mechanistically, the AMPK pathway can negatively regulate PD-1 expression through the HMGCR/p38 MAPK/GSK3β signaling axis, providing a rationale for combining AMPK activators with anti–PD-1 antibodies to enhance therapeutic efficacy (Pokhrel et al., 2021). However, the application of ICIs in sepsis remains in its infancy. Unlike cancer, the immune state in sepsis is highly dynamic and unstable, and excessive checkpoint blockade could precipitate uncontrolled inflammatory rebound, worsening tissue injury. A retrospective study even reported higher one-year mortality (22.2%) among septic patients receiving combined PD-1 and CTLA-4 inhibition compared with monotherapy, underscoring the potential risks (Yu et al., 2025). At present, no ICIs have been approved for sepsis treatment, and their safety, efficacy, and optimal timing require validation in rigorous clinical trials (Hensler et al., 2022). In addition to PD-1 and CTLA-4, other immune checkpoints such as TIM-3, OX40, and 2B4 have also been implicated in sepsis-related immune dysregulation and represent additional potential therapeutic targets (McBride et al., 2021). The upregulation of these checkpoint molecules is a hallmark of the late immunosuppressive phase, which is precipitated by the earlier inflammatory cascade. Emerging evidence suggests that sustained signaling through pathways such as TLR4 can contribute to a state of immune exhaustion. The PI3K/Akt pathway, negatively regulated by PTEN, has been shown to intersect with PD-1 signaling, suggesting that the initial loss of PTEN-mediated control over inflammation may inadvertently foster an environment conducive to subsequent T cell dysfunction and checkpoint upregulation.

### 4.3 Metabolic pathway-focused interventions

The functional dysregulation of immune cells in sepsis is closely intertwined with pronounced metabolic reprogramming, making the modulation of cellular metabolism an attractive therapeutic strategy. Many of these metabolic shifts are orchestrated by the PI3K/Akt/mTOR pathway, which is tightly regulated by the TLR4/PTEN axis. As detailed in Section 1.1, PTEN loss in sepsis removed a crucial brake on this anabolic signaling. Consequently, sepsis disrupts the equilibrium between energy-sensing AMPK and mTOR, resulting in abnormal immune metabolic activity. Studies have shown that AMPK activators (e.g., AICAR) can correct macrophage autophagy defects induced by BCG through the AMPK-mTOR-ULK1 axis, thereby enhancing their bactericidal and immunomodulatory functions (Cheng et al., 2025). These findings position AMPK activators as potential countermeasures to the metabolic consequences of PTEN dysfunction. Preclinical models suggest modulating metabolic pathways like AMPK/Akt/mTOR can improve outcomes (Zhang et al., 2025). Recently, the GAS6/Axl-AMPK signaling pathway has been established as another promising target, as its modulation can simultaneously ameliorate inflammation and improve organ function (Lei et al., 2024). Conversely, mTOR inhibitors such as rapamycin regulate the TFEB-mediated autophagy-lysosomal pathway, promoting the clearance of damaged organelles and pathogens. This process may protect against proteotoxic stress and enhance infection control in sepsis (Xu et al., 2022). A key challenge facing metabolic intervention efforts lies in the substantial variation in metabolic profiles observed across the stages of sepsis, particularly when contrasting the hypermetabolic/inflammatory stages and the late hypometabolic/immunoparalytic stages, with this issue being further compounded by variability among immune cell subsets within the same stage. As a result, precise metabolic modulation must be tailored to each patient’s specific immunometabolic phase and cellular context to minimize the potential for adverse outcomes (Louaguenouni et al., 2025; Vandewalle and Libert, 2022).

The rationale, current status, and main challenges of these immunomodulatory strategies are summarized in **Table 3**.

**Table 3:** Summary of key immunomodulatory therapeutic strategies in sepsis.

Class	Rationale	Clinical status	Key challenge
Anti-Cytokine (e.g., anti-TNF-α)	Target upstream driver of cytokine storm	Failed in unselected RCTs	Timing, heterogeneity
Immune Checkpoint Inhibitors	Reverse T cell exhaustion	Early-phase studies; no approval	Inflammatory rebound risk
Metabolic Modulators	Correct immunometabolic reprogramming	Predominantly preclinical	Stage- and cell type-specificity

### 4.4 Challenges in translating mechanistic insights into clinical success

Despite extensive preclinical evidence supporting diverse immunomodulatory targets, their translation into effective sepsis therapies has remained largely unsuccessful. This translational gap arises from several interrelated factors. (1) Patient heterogeneity: The absence of endotype-based stratification has likely diluted treatment effects in unselected patient populations (Nakamori et al., 2021). 2) Critical timing: The dynamic trajectory of sepsis immunopathology renders therapeutic efficacy highly time-dependent. For example, anti-inflammatory interventions may prove ineffective or even detrimental if administered after the onset of immunoparalysis (Nykänen et al., 2024; Samuelsen et al., 2024). 3) Suboptimal trial design: Historical clinical trials frequently employed broad inclusion criteria and relied on mortality as the primary endpoint—an outcome measure that may fail to capture therapeutic benefits within specific immunological subgroups. Future investigations should adopt adaptive trial designs, biomarker-guided patient enrollment, and endpoints that reflect immune reconstitution (Van Nynatten et al., 2025; Vissichelli et al., 2021). Recognizing these challenges is critical for developing the next generation of precision immunotherapy trials in sepsis.

## 5 Clinical translation of precision treatment strategies

### 5.1 Single-cell sequencing-based immune endotyping technologies

Traditional bulk sequencing conceals cellular heterogeneity, whereas single-cell RNA sequencing (scRNA-seq) resolves the intricate immune landscape of sepsis at single-cell resolution. This technology enables the unbiased identification of novel, rare, and functionally specialized immune cell subsets, delineating their transcriptional signatures and dynamic states. Such insights provide a robust foundation for stratifying patients into distinct endotypes with defined mechanistic bases and therapeutic implications (as summarized in **Table 4**). This technology can identify novel, rare, or functionally specialized immune cell subsets in an unbiased manner and delineate their transcriptional signatures and dynamic states. Application of scRNA-seq to peripheral blood mononuclear cells (PBMCs) from septic patients has revealed significant alterations in both innate and adaptive immune populations. For instance, it reveals altered differentiation and activation states in monocyte/macrophage subsets that correlate with disease severity (Ning et al., 2023; Zhao et al., 2023). Importantly, scRNA-seq data demonstrate a widespread acute suppression of the adaptive immune system (T and B cells), characterized by attenuated T cell receptor signaling, limited clonal expansion, and reduced effector function, all of which are factors that critically contribute to impaired host defense (Feng et al., 2025). Beyond conventional classification, scRNA-seq facilitates the identification of functional endotypes based on distinct transcriptional signatures. For example, discovery of a “persistently hyperinflammatory” (RAI) endotype marked by specific myeloid subsets exhibiting sustained inflammatory signaling offers direct molecular targets for precision immunomodulation tailored to defined patient subgroups (Murao et al., 2024; Wu et al., 2023). These endotypes correlate with distinct underlying molecular mechanisms (Section 2), biomarker profiles, and therapeutic vulnerabilities, providing a practical framework for precision therapy (as summarized in **Table 4**).

**Table 4:** Immune endotypes in sepsis: linking mechanisms, biomarkers, and targeted therapies.

Immune endotype	Core immunopathological mechanism	Key biomarkers/Features	Potential targeted interventions
Early Hyperinflammatory	Excessive TLR4/NF-κB signaling, cytokine storm (TNF-α, IL-6)	High PCT, CRP, IL-6; mHLA-DR^hi^; Neutrophil hyperactivity	Anti-TNF-α, IL-6R antagonists, TLR4 inhibitors, short-course corticosteroids
Late Immunosuppressive	Immune cell exhaustion (PD-1/PD-L1↑), metabolic reprogramming (AMPK/mTOR dysregulation), apoptosis	Lymphopenia; mHLA-DR^low^; High PD-1 expression on T cells	Immune checkpoint inhibitors (anti-PD-1), IL-7, GM-CSF, AMPK activators
Mixed/Transitional	Coexistence of inflammatory and suppressive signals	Dynamic changes in PCT/CRP ratios; presence of both inflammatory cytokines and immunosuppressive cells	Staged therapy guided by dynamic immune monitoring

### 5.2 Dynamic immune monitoring-guided staged therapy

Dynamic immune monitoring emphasizes serial evaluation of a patient’s immune status through integrated methodologies that combine conventional biomarker measurements (e.g., PCT, CRP, HBP), flow cytometric analysis of lymphocyte subsets (e.g., CD4^+^/CD8^+^ T cell counts), and functional markers such as monocyte human leukocyte antigen–DR (mHLA-DR) expression (Darden et al., 2021; Robey et al., 2024). Establishing such a monitoring framework enables real-time tracking of a patient’s immunological trajectory from the hyperinflammatory “cytokine storm” phase to the subsequent immunosuppressive phase, thereby guiding “staged” or “adaptive” therapeutic strategies. The core principle is to apply distinct and sometimes opposing interventions according to the current immune phase. For instance, dynamic monitoring may identify patients exhibiting a “persistently hyperinflammatory” endotype, for whom cautious corticosteroid use within a narrowly defined therapeutic window could mitigate excessive inflammation. In contrast, the same treatment could prove detrimental in patients experiencing profound immunosuppression, where immunostimulatory interventions (e.g., IL-7, GM-CSF, or ICIs) may be more appropriate (Wu et al., 2023). Physical immunomodulatory techniques, such as leukocytapheresis, have been reported to exert bidirectional effects, simultaneously suppressing hyperinflammation and enhancing immune responsiveness, thus leading to improved hemodynamics. However, their efficacy is highly dependent on precise timing and patient selection guided by dynamic immune assessment (Zhou et al., 2022). Thus, treatment decisions are shifting toward individualized, immune phenotype-driven strategies (Zhou et al., 2022).

### 5.3 Machine learning-driven personalized treatment models

The complex, high-dimensional, heterogeneous, and dynamically evolving data landscape of sepsis provides an ideal setting for the application of machine learning (ML) algorithms, which hold considerable promise for advancing personalized disease management. ML techniques can integrate diverse sources of information, including clinical parameters, vital signs, high-throughput omics data, and temporal biomarker trajectories to develop predictive models and clinical decision-support systems. A major application of these models lies in predicting a patient’s potential response to specific therapeutic interventions. For example, by analyzing multidimensional features collected at hospital admission, ML can estimate the likelihood that a patient will benefit from corticosteroids, vasopressors, or particular antibiotic regimens, thereby enabling data-driven and objective drug selection (Zhang et al., 2025; Zheng et al., 2022). In addition to predicting therapeutic efficacy, ML models can analyze continuous clinical data streams to identify the optimal timing for initiating or discontinuing specific immunomodulatory treatments, addressing the critical challenge of therapeutic timing in sepsis. Moreover, the application of unsupervised learning algorithms to large-scale clinical datasets can reveal novel patient subgroups (endotypes) characterized by distinct prognostic and therapeutic profiles, thereby transcending traditional classification frameworks and laying the groundwork for redefining sepsis taxonomy and precision treatment strategies (Chen et al., 2023). Proof-of-concept computational modeling studies have further demonstrated this potential. For example, computational models can identify patient subgroups likely to benefit from specific interventions, such as immune checkpoint inhibitors in select cases of bacterial sepsis (Chen et al., 2023). Another important application of ML involves guiding more precise molecularly targeted interventions. By analyzing individual molecular and signaling profiles, ML can help identify patients more likely to benefit from specific modulators of the TNF signaling pathway, for instance, those suited to TNFR2 agonists versus TNFR1-specific antagonists (Su et al., 2022). As gene editing and cell engineering technologies advance, ML models may eventually guide the development of customized cell-based therapies tailored to each patient’s immune phenotype, such as engineered CAR-macrophage (CAR-M) therapies, thereby pushing the boundaries of personalized medicine (Cajander et al., 2024; Li et al., 2025; Ye et al., 2024). The convergence of these emerging technologies collectively accelerates the evolution of sepsis management toward true precision medicine (Cajander et al., 2024).

## 6 Current challenges and future directions

### 6.1 Controversy surrounding the timing of immunomodulatory therapy

The optimal timing of immunomodulatory interventions in sepsis remains uncertain and represents a major contributor to clinical trial failures. Sepsis immunopathology is inherently dynamic and heterogeneous, with patients frequently exhibiting concurrent hyperinflammatory and immunosuppressive features. Administration of anti-inflammatory agents at late stages may exacerbate immunosuppression, whereas early use of immunostimulatory therapies can intensify the inflammatory response (Robey et al., 2024; Slim et al., 2024). Future progress will depend on the implementation of dynamic biomarker monitoring to guide real-time therapeutic decisions.

Furthermore, the optimal timing and nature of immunomodulatory interventions may differ according to the causative pathogen. Bacterial sepsis typically manifests with a pronounced early hyperinflammatory phase, potentially providing a therapeutic window for targeted anti-cytokine interventions. In contrast, viral infections such as severe influenza and COVID-19 involve a more intricate interplay between viral replication and host immune responses, in which premature immunosuppression may be harmful and the efficacy of immunostimulatory approaches requires cautious evaluation. Fungal sepsis, which frequently arises in immunocompromised hosts, is characterized by a predominant immunosuppressive state from the onset, thereby favoring the application of immunostimulatory strategies. This pathogen-specific heterogeneity highlights the necessity of integrating rapid microbiological diagnostics with immune phenotyping to inform therapeutic decision-making (Jain et al., 2024).

### 6.2 The challenge of stratified therapy in a heterogeneous patient population

The extreme heterogeneity of sepsis patients remains a major barrier to precision therapy. Traditional binary classifications (e.g., “pro-inflammatory” vs. “immunosuppressed”) oversimplify the complex immune landscape. This oversimplification has contributed to the repeated failure of immunomodulatory clinical trials designed around such coarse stratification strategies (Shankar-Hari et al., 2024). Interindividual variability in sepsis arises from differences in immunometabolic regulation, epigenetic programming, and microbiome composition. While the integration of multi-omics data—including genomics, transcriptomics, proteomics, and metabolomics—can facilitate the delineation of distinct patient subtypes, translating these findings into clinically applicable tools remains a significant challenge. Furthermore, the relationships between routine biomarker trajectories and therapeutic responses are often ambiguous, thereby limiting progress toward true personalization. Machine learning approaches that integrate multi-omics and clinical datasets represent a promising avenue to overcome these limitations and enable precision-guided therapy.

### 6.3 Application prospects of novel bioengineering technologies

**Figure 2:**
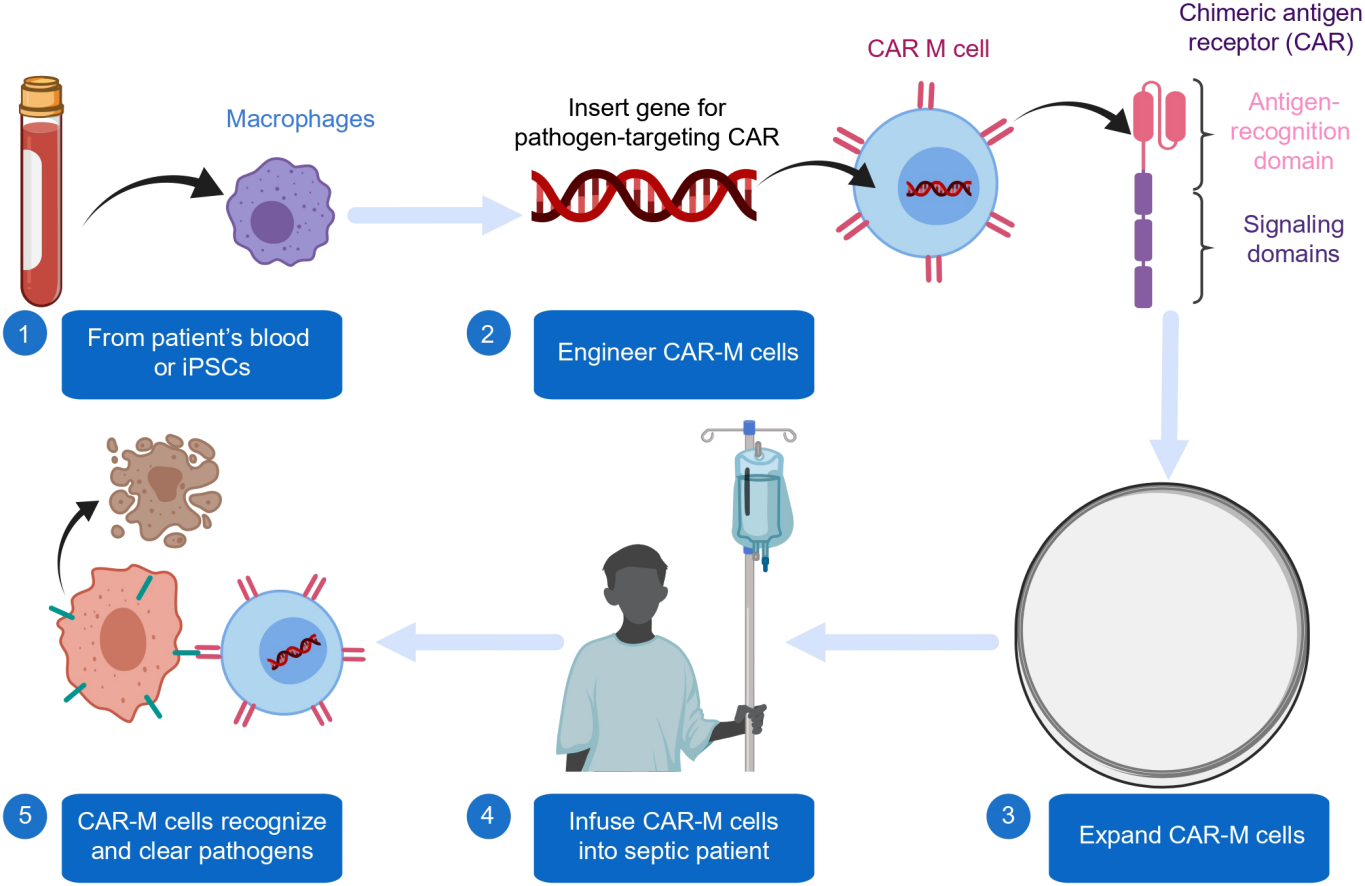
Pathogen-specific innate immune signaling pathways in early sepsis. This schematic illustrates the initial hyperinflammatory response characteristic of sepsis, emphasizing how distinct classes of pathogens are recognized by specific pattern recognition receptors (PRRs) to initiate convergent proinflammatory signaling cascades. Bacterial components (e.g., lipopolysaccharide, LPS) are primarily detected by the TLR4/MD2 complex on the cell surface. Viral nucleic acids (e.g., double-stranded RNA, dsRNA, or single-stranded RNA, ssRNA) are sensed by intracellular receptors such as TLR3 and RIG-I. Fungal cell wall components (e.g., β-glucan) engage receptors including Dectin-1 and TLR2. Despite these distinct upstream triggers, signaling converges through adaptor proteins such as MyD88 and TRIF, leading to the activation of key transcription factors, notably NF-κB and IRF3/7. This coordinated signaling drives the production of a potent cytokine and interferon milieu (e.g., TNF-α, IL-6, IL-1β, and type I IFNs), culminating in the so-called “cytokine storm.” The figure highlights the mechanistic basis of immune heterogeneity, illustrating how different etiologies—bacterial, viral, or fungal—elicit distinct signaling intensities and temporal dynamics through these dedicated pathways, thereby underscoring the necessity of pathogen-informed diagnostic and therapeutic strategies.

Chimeric antigen receptor–macrophage (CAR-M) therapy leverages the intrinsic tropism of macrophages toward sites of infection and their phagocytic and antigen-presenting capacities. Preclinical studies have demonstrated that engineered CAR-M cells can selectively target pathogens for clearance while modulating the immune microenvironment through cytokine secretion (e.g., IFN-γ), thereby exerting both antimicrobial and immunomodulatory effects (Kang et al., 2021; Song et al., 2022)(see **Figure 2**). In experimental sepsis models, engineered macrophages have been shown to fine-tune TLR4 signaling and other innate immune signaling pathways, effectively clearing pathogens while minimizing collateral inflammatory injury (Lin et al., 2021; Song et al., 2022). Despite these promising findings, CAR-M therapy faces several challenges, including complex manufacturing processes, high production costs, and concerns regarding the persistence, stability, and phenotypic plasticity of the engineered cells within the dynamic *in vivo* immune environment (Cajander et al., 2024; Chen et al., 2023). To address these limitations, the development of induced pluripotent stem cell (iPSC)-derived CAR-M cells (CAR-iMacs) has emerged as a potential “off-the-shelf” solution that could reduce production time and cost while enhancing scalability (Chin and Elisseeff, 2022; Su et al., 2022). Future research should prioritize improving the functional stability and adaptability of CAR-M therapies in the complex immunological milieu of sepsis and exploring their integration with complementary technologies, such as nano-immunomodulators and bioengineered delivery platforms, to enable safe and effective clinical translation (Feng et al., 2025; Giamarellos-Bourboulis et al., 2024; Liu et al., 2025).

However, the application of chimeric antigen receptor–macrophage (CAR-M) therapy in sepsis remains largely theoretical and faces substantial translational challenges. The principal limitations include the complex and costly ex vivo manufacturing process, the risk of off-target activity or excessive inflammatory responses, and the complete absence of clinical safety or efficacy data in septic patients. Most critically, the time required for the generation of autologous CAR-M cells is fundamentally incompatible with the acute, rapidly evolving course of septic shock. Although “off-the-shelf” platforms such as CAR-induced macrophages (CAR-iMacs) present a potential solution, their stability and therapeutic efficacy within the dysregulated immune environment of sepsis remain unverified. For broader clinical applicability, alternative cell-based approaches—such as mesenchymal stromal cells (MSCs), which exhibit pleiotropic immunomodulatory properties and are already under evaluation in advanced clinical trials—may represent a more immediately feasible option (Ye et al., 2024). Future investigations must rigorously address these challenges before CAR-M therapy can be considered a viable therapeutic strategy.

A paramount challenge for autologous CAR-M therapy in sepsis is the stark mismatch between production timelines and disease kinetics. The conventional process—isolating a patient’s monocytes, genetically engineering them, expanding the CAR-M population, and validating their safety and function—is a protracted endeavor, typically spanning several weeks. This timeline is fundamentally incompatible with the management of septic shock, where the critical window for intervention is measured in hours to days. Consequently, the clinical feasibility of CAR-M therapy for acute sepsis is entirely contingent upon the development of a rapid, ‘off-the-shelf’ approach. This reality underscores the critical importance of advancing allogeneic platforms, such as those utilizing iPSC-derived CAR-iMacs. These cells can be pre-manufactured, quality-controlled, and banked for immediate administration, analogous to the use of frozen plasma or packed red blood cells in a transfusion service, thereby bridging the temporal divide between sophisticated cell engineering and emergent clinical need.

Recent advances have further propelled progress in this field. Notably, the development of “signal-switch” chimeric antigen receptor–macrophages (CAR-Ms), which can dynamically modulate their inflammatory activity *in situ*, represents a substantial step toward safer and more adaptable cellular therapies for complex inflammatory conditions such as sepsis (Cao et al., 2025).

## 7 Conclusion

Sepsis presents a formidable therapeutic challenge, not due to a lack of potential targets, but because of the profound temporal and interindividual heterogeneity of its immunopathology. The repeated failure of monolithic immunomodulatory strategies underscores that the era of “one-size-fits-all” treatment is over. The path forward lies in a precision medicine framework that dynamically aligns therapeutic interventions with a patient’s evolving immune status. This necessitates a fundamental shift from diagnosing a syndrome to defining an individual’s immunological endotype.

This shift is being enabled by the integration of advanced technologies. Single-cell sequencing and high-dimensional immune profiling define mechanistically driven endotypes, while machine learning models translate these insights into bedside decisions. A deep understanding of core pathways (e.g., TLR4/PTEN, IDO1) provides the molecular lexicon for selecting targeted therapies. Looking ahead, the convergence of immunology and bioengineering—exemplified by “off-the-shelf” cell-based therapies like CAR-Macrophages—promises to blur the line between diagnosis and therapy. Despite persistent challenges in timing and safety, the strategic integration of deep phenotyping, data analytics, and novel platforms offers a path toward personalized sepsis management and breaking the long-standing therapeutic impasse.
